# Small business managers and Covid-19—The role of a sense of coherence and general resistance resources in coping with stressors

**DOI:** 10.1371/journal.pone.0265029

**Published:** 2022-03-18

**Authors:** Josefine Hansson, Bodil J. Landstad, Stig Vinberg, Marianne Hedlund, Åsa Tjulin

**Affiliations:** 1 Department of Health Sciences, Faculty of Human Sciences, Mid Sweden University, Östersund, Sweden; 2 Unit of Research, Education and Development, Östersund Hospital, Östersund, Sweden; 3 Department of Social Work and Health Sciences, Norwegian University of Science and Technology, Trondheim, Norway; Universiti Pertahanan Nasional Malaysia, MALAYSIA

## Abstract

**Background:**

The response of small business managers to an external event such as the pandemic can have a profound effect on the work environment, health and well-being for themselves and their employees. Previous research on small business managers during the pandemic has mainly focused on traditional pathogenic effects, and there is a lack of studies looking at the issue from a salutogenic health promotion perspective. The aim of this study is to explore whether a sense of coherence and general resistance resources were experienced by small business managers in Sweden and Norway during the Covid-19 pandemic.

**Methods:**

A qualitative design was applied through exploratory interviews with 16 managers of small businesses in Sweden and Norway. A content analysis of the interviews was conducted using the sense of coherence concept by Aaron Antonovsky, with the three main components of comprehensibility, meaningfulness and manageability acting as a conceptual framework for the analysis process.

**Results:**

Within the three main sense-of-coherence categories, six general resistance resources were identified as being important for the managers to handle uncertainty during the pandemic. These were understanding rules and regulations, social support, optimism, proactivity, problem-solving and flexibility and cooperation.

**Conclusions:**

The small business managers handled the pandemic in a way that worked well in their contexts, and the pandemic generally did not have a negative effect on their businesses or themselves. A salutogenic approach, through which the managers focused on identifying and using resources, was an important factor for managing stressors and adversity during the pandemic. Hence, the concept of salutogenesis may be used as an intervention to foster better health in small businesses, both at a personal and organisational level in order to handle future challenges effectively.

## Introduction

Small businesses are highly important job and income generators and are considered vital in national and local economies [1, 2]. They are conducive to competitiveness, economic growth and employment in Europe, and they stimulate a sense of entrepreneurship and innovation. Many businesses around the world are facing a significant impact from the Covid-19 pandemic, [3] with the transport, tourism and hospitality sectors being among the most affected [4]. Compared to larger businesses, the effect on small businesses has generally been more severe due to their limited human, financial and technical resources [5]. In addition, the degree of government financial aid, in combination with changes in the supply chain, may strongly affect the operations and survival of small businesses [5]. A large European survey conducted early in the pandemic reported that small business managers had a worse domestic financial situation and significantly higher job insecurity compared to waged workers [6]. Similarly, a survey looking at small business managers in 23 countries, found that 61% of the managers perceived their businesses to be under threat due to a decrease in trading activities in the early days of the pandemic. It was also noted that mental well-being dropped by 12%, creating another threat to their businesses [7]. This aligns with results from a mixed-method study [8], which found lower scores for well-being among Swedish small business managers compared to before the pandemic. Even though managers of small businesses have faced significant challenges during the pandemic, studies have also reported a resilience in managers in terms of finding new business solutions, being agile and having a positive long-term outlook for their businesses [7–9].

The response of the managers of small businesses to an external event such as the Covid-19 pandemic can have a profound effect on the work environment, health and well-being for themselves and their employees. Even before the pandemic, smaller businesses offered fewer health and safety programs and fewer benefits for workers when compared with larger businesses [10]. One explanation for this may be a lack of time, resources and knowledge to identify risks in smaller businesses [9, 10]. However, previous research has shown that small-business managers wish to incorporate workplace health management but often lack the knowledge and resources to do so [11, 12].

### Study context

Approximately 99% of Swedish businesses are considered as small businesses with up to 49 employees [13], while the corresponding figure for Norway is 98% [14]. Sweden and Norway have similar population, educational and political systems but their governments have had different approaches to the Covid-19 pandemic. For instance, Norway imposed a lockdown and closed everything except grocery stores and pharmacies in the early days of the pandemic [15]. Sweden on the other hand kept most institutions and facilities open but introduced restrictions, and group gatherings were limited to a maximum of 49 people [15, 16].

Swedish and Norwegian small businesses have been affected by the pandemic and demand for many of their services has declined [8, 17]. In both Sweden and Norway, the governments implemented measures to support small business managers and their employees during the crisis. In Sweden, support was given in the form of subsidized rent to the most-affected businesses, as well as bridge loans to small business start-ups [18]. Norwegian support included bank loans, cash support and new layoff rules with the government contributing most of the unemployment benefits [17]. However, governmental support was only used by some of the managers as several stated that they did not meet the requirements for application or that they were unsure of their eligibility [8, 19].

Although there are some studies that focus on small business managers during the Covid-19 pandemic, these studies have mainly examined traditional pathogenic effects such as burnout, sickness presenteeism, detrimental health effects and work stressors [9, 20–22]. Studies on small business managers from a salutogenic health promotion perspective are lacking. A salutogenic approach may help to identify important resistance and health-promoting resources, which may be beneficial for small business managers to counteract stressors during adversity and in everyday life. The aim of this study is to explore whether a sense of coherence (SOC) and general resistance resources (GRRs) were experienced by small business managers in Sweden and Norway during the Covid-19 pandemic.

### Conceptual framework

#### Sense of coherence

SOC is the main concept of the Salutogenic model and is considered to be a determinant of health. Antonovsky [23] created this conceptual framework to understand why some people thrive in the face of adversity, whereas others succumb to it. According to Antonovsky [24] a person with a strong SOC is cognitively and emotionally capable of understanding problems and is willing to face them. In contrast, a weak SOC decreases an individual’s ability to cope with difficult situations effectively. The SOC consists of three components: comprehensibility, meaningfulness and manageability. Comprehensibility is a measure of the ability to perceive incidents and circumstances as structured and understandable. Meaningfulness is the belief that challenges and demands are worthy of investment. Manageability is the ability to cope with difficult situations [23].

GRRs are another main component of the framework of the salutogenic model [23]. GRRs encompass the resources available to any of us at a given time and can include attitudes and beliefs, or be of social, material and physical character. Individuals with a high SOC perceive the world as more comprehensible, manageable and meaningful, and are consequently better at understanding stressors, accepting the challenge to handle them, and identifying GRRs to deal with these stressors [24, 25].

It was previously thought that a person’s SOC was relatively fixed by the age of 30 [23]. However, more recent studies have found that it increases slightly with age [26] and may decrease through negative or drastic life events [27]. Different interventions have also been found to influence SOC levels. Such interventions include mindfulness [28], activities that facilitated reflection on the SOC [29], and movement, sports and games [30]. Furthermore, a supportive social environment is also an important factor in developing and maintaining a strong SOC [31, 32].

#### Sense of coherence in work-related research

Different theoretical models have been used to examine the associations between working conditions and stress-related outcomes. The Job Demand-Control Model (JDC), is a commonly studied theoretical model that suggests that high demands and a low level of control in the workplace increase job strain, which in turn may lead to stress-related ill-health [33]. Another common model is the Job Demands-Resources model (JD-R), which suggests that job resources can buffer the impact of job demands on strain [34]. The thing that distinguishes SOC from the traditional job strain models is its focus on the individual’s own ability to mobilise resources to cope with stressors and manage tension [35]. It is known that individual characteristics may affect a person’s way of handling as well as perception of, and scope of control [36]. One study found that individuals with a strong SOC cope more effectively with strain in the work environment and the authors stated that individual factors such as SOC should be included in investigations of the effect of work environment factors on stress and well-being [37]. Other research on work-related SOC has also shown that a strong SOC is correlated with health-related quality of working life [38], high levels of work engagement [39, 40] and less work-related stress [41]. SOC has successfully been incorporated into other qualitative studies, mainly in healthcare settings [42–44]. A recent systematic review provided evidence that a SOC helps to protect against a depressive state, burnout and job dissatisfaction among nurses, and that it is important to build a healthy work environment to foster the SOC [45].

To our knowledge, no qualitative studies have been carried out regarding SOC and small business managers. However, the Life Orientation Questionnaire (SOC-13) by Antonovsky [24], which is used to measure the SOC, has been used in quantitative studies. Recently, Küsel, Schultz and Rankhumise [46] used the questionnaire when investigating whether conceptual entrepreneurial competencies predicted the SOC in female small business managers in South Africa. Conceptual competencies are related to specific behaviours of the entrepreneur such as a persuasive ability, communication and interpersonal skills. The study reported a significant positive correlation between the SOC and conceptual entrepreneurial competencies. However, the study sample was small (n = 241) and most businesses were start-ups which may have influenced the results. Additionally, in a literature review of small business managers’ health, Torrés and Thurik [47] state that salutogenic factors and their effects on the health of small business managers are under-researched and that an extensive research agenda on the topic is required.

#### Salutogenesis and coping with Covid-19

The Covid-19 pandemic has required people all over the world to adjust to a new chaotic reality [48]. Recent studies highlight the important role of a SOC in coping with the pandemic to mitigate acute and chronic stressors [49]. For instance, Mana et al. [50] found that coping resources were significant in predicting anxiety and mental health in individuals from different countries during the pandemic, and that SOC was the main predictor of these two reactions. The authors concluded that a person’s ability to view life as comprehensive, manageable and meaningful during a pandemic was the most important coping resource in different national and social contexts. Similar results were found in another study during the pandemic where SOC was found to have a moderating effect on the link between illness experiences and psychological well-being in the general population. Specifically, lower levels of SOC in individuals who knew someone diagnosed with Covid-19 resulted in lower levels of psychological well-being [51]. Furthermore, a large cross-sectional study during the Covid-19 pandemic in Germany found that a strong SOC was associated with less anxiety and depression symptoms, and that higher levels of SOC buffered the impact of Covid-19 stressors on general health [52]. In regards to managerial research, a study focusing on exploring SOC in South African managers from different organisations found that the managers scored in the higher and medium range of SOC. They perceived the situation of the pandemic as understandable, manageable and meaningful, enabling the activation of their resilience. It was assumed that managers participating in the research did not suffer much during the pandemic [53].

## Methods

### Study design

The research was designed as a descriptive deductive study using exploratory interviews with small business managers in Sweden and Norway. A qualitative design was deemed appropriate as the aim was to gain an understanding of how the small business managers made sense of their experiences during the pandemic. A content analysis of the interviews was conducted, in which the SOC concept by Antonovsky [24] acted as the conceptual framework for the analysis process.

### Participants

Sixteen managers were purposively recruited by two occupational health service providers in Norway and Sweden, selected from among their customers and through advertisements in newspapers. The inclusion criteria were small businesses with less than 20 employees representing different types of services in the private sector. Further criteria were that the businesses employed people of both genders and that they were located in comparable geographic regions in Sweden and Norway. As seen in Table 1, half of the 16 managers were women. Eight managers had university-level education, the others had either upper-secondary, vocational or high-school education. The age of participants varied between 38 and 73 years. The majority, thirteen managers, were employed in the service industry, and the remaining three were in the building and construction industry.

**Table 1:** Characteristics of the participants.

Country	Sweden	Norway
**Managers, total**	9	7
**Gender**		
• Male	4	4
• Female	5	3
**Age**		
• < 40	1	0
• 41–50	2	4
• 51–60	3	2
• > 61	3	1
**Education**		
• High school	2	0
• Vocational training school	2	2
• Upper secondary school	1	1
• University	4	4
**Civil status**		
• Married/cohabiting	8	6
• Single	1	3
**Industry**		
• Building & mechanical construction	2	1
• Service industry (Kindergarten, wellness services, food industry, media, animal husbandry, sales businesses)	7	6

### Data collection

Data was collected through individual interviews via video link or telephone between January and March 2021. One experienced researcher (Author 5) conducted the interviews in Sweden together with a PhD student (Author 1) who asked supplementary questions at the end of the interviews. The Norwegian interviews were performed by two experienced researchers (Author 2 and Author 4). A semi-structured interview guide was used for the interviews [54]. The topics covered the managers’ health, work environment and leadership during the Covid-19 pandemic. Each interview lasted an average of 50 minutes and was audio-recorded and transcribed verbatim after the interview. A pilot interview was performed with one small business manager in another region in Sweden to test the relevance of the questions.

### Data analysis

A content analysis as described by Elo and Kyngäs [55] was used for the data analysis. This type of analysis method is suitable when conducting exploratory work in an area where not much is known [56]. The SOC theory [24] was used as the conceptual framework. Initially the interview transcripts were read several times to make sense of the whole. A categorization matrix with the pre-set categories of comprehensibility, meaningfulness and manageability was then created using the NVivo qualitative analysis package (1.4.1). Once the categorization matrix was developed, all the data was reviewed for content, and condensed meaning units were coded in line with the identified categories [55]. Meaning units were coded into the comprehensibility category when managers discussed if and how they made sense of information or situations during the pandemic. When statements concerned their motivation and desire to cope with the stimuli encountered, the meaning units were coded into the meaningfulness category. Meaning units were coded into the manageability category when managers discussed the degree to which they felt that there were resources at their disposal during the pandemic. In addition, when they mentioned their ability to cope and solve problems and their willingness to invest time and energy to solve those, the meaning units were assigned to this category.

The coded meaning units in each category were then grouped and categorized according to their meanings and similarities into six sub-categories which were based on GRRs (Table 2). The sub-categories with the majority of codes were chosen for further analysis. These were understanding rules and regulations, social support, optimism, proactivity, problem-solving and flexibility and cooperation.

**Table 2:** Coding framework based on the sense of coherence categories.

Category	Sub-category	Code	Meaning unit
**Comprehensibility**	**Understanding rules and regulations**	Understanding and discussing information	“We had meetings with the staff, to make sure that everyone knew what was going on and how to behave and so on (…). We felt safe then. We didn’t actually feel any great stress.” (IP 9)
	**Social support**	Discussed the situation with others	“So the general situation is a little more uncertain and we talk about it every day, at coffee in the morning, at lunch and things like that.” (IP 3)
**Meaningfulness**	**Optimism**	Feeling of hope	“I did not experience the period to be too stressful (…). I still felt that there is hope, you have to eat somewhere so we have to come back. And get sales started again.” (IP 7)
	**Proactivity**	Worked proactively	“Instead of us sitting passively and waiting, for a deal or a contract, we brought people back and sat down and started to assess the market, showed ourselves as offensive and at the same time addressed the requests that came our way. (…) It has borne fruit in retrospect.” (IP 18)
**Manageability**	**Problem-solving**	Changed way of working	“And something we’re going to do soon, is that’s we’re going to start importing a little Italian pasta (. . .). And it is possible to sell to private individuals as well, and not just to restaurants." (IP 9).
	**Flexibility and cooperation**	Held digital meetings	“Previously we’ve been travelling and visiting suppliers and suppliers have come and visited us and there has been quite a lot of travelling. I’ve had a few meetings outside of the city and instead of having physical meetings, we’ve had Teams meetings (…). That’s pretty much time-saving.” (IP 16)

### Ethics

Ethical application was approved by Sweden’s Ethical Review Board (2020–05223). The participants gave written consent to participate in the study and raised no objections from an ethical point of view. All of the data were properly stored according to the Swedish Act on Ethical Review of Research Involving Humans (SFS 2003:460).

## Findings

Within the three main SOC categories, six general resistance resources were identified in the material that were deemed to be important for the managers to handle uncertainty during the pandemic.

### Comprehensibility

The first category identifies small business managers’ level of comprehensibility i.e. that incidents and circumstances are structured and understandable. The category is built on the general resistance resources *understanding rules and regulations* and *social support*. These represent how the managers received and made sense of information during the pandemic.

#### Understanding rules and regulations

Despite the fact that Sweden’s public health responses to the pandemic were less restrictive and were introduced slower than those in Norway, the managers had a similar experience of the information flow regarding restrictions and rules of conduct. The managers in both countries stated that they had to actively look for information regarding restrictions and regulations, and some of the channels through which it was obtained included governmental websites, press conferences on TV and via the media in general. Initially, some of the managers felt that the specific information for small businesses was scarce and difficult to find, which resulted in incomprehensibility of the situation. However, as more information gradually became available the situation became clearer, and they felt confident in adapting their businesses in accordance with the restrictions and recommendations. A Swedish manager mentioned that it would be beneficial going forward to equip new managers with knowledge on where to find information and material in case of similar future events. She stated that over the years she had learnt where to find information and found it simple to do so. Moreover, some experienced managers stressed the importance of setting aside time to really try to familiarise themselves with the information available in order to feel confident in their actions. By taking the time to go through the information available they increased their comprehensibility of the situation.

*“What is important, I think, is to set aside time to really take on what’s happening and what information is available. So you don’t just close your eyes and think, "Wow, how terrible this is, what am I supposed to do?" without really grasping the information. What information is there, where can I find my information?” (IP 9)*

#### Social support

Social support from colleagues and family helped the managers deal with stressors that arose during the pandemic. They regularly discussed and solved issues together with employees at meetings, as well as in more informal contexts such as during coffee breaks and conversations in the corridors–all with recommended distance. The support of others created a feeling of belonging and togetherness and helped them cope with the situation more easily. Several of the Norwegian managers participated in professional networks that were offered online by their local municipality where they exchanged experiences regarding the pandemic. This was seen as very valuable, and by discussing these experiences with others in the same profession they increased their understanding of the situation and gained insight into how others solved different tasks.

*“We discuss how we do things, how we understand the rules, how you look at it and how… It’s great support to have as a leader.” (IP 11)*

One of the managers said that he discussed matters with his employees but that there were certain topics that were unsuitable to bring up with them. For instance, matters regarding the workforce. *“We talk a lot in the group*, *of course we do*. *But my biggest “sounding board” is probably at home*, *my wife*. *Where I get to discuss a little*. *A way to tackle these situations*.*” (IP 1)*

In summary, the data in this category showed the importance of understanding information and regulations during the pandemic in order to feel confident in decisions and actions. Discussing and exchanging information with others helped to gain a better understanding of the situation.

### Meaningfulness

The second category identifies whether the managers were able to find meaning in the situation, i.e. the extent to which an individual believes that life makes sense emotionally, and that one possesses the motivation and desire to cope with any stimuli encountered. This category is built on the general resistance resources *optimism* and *proactivity*, representing how the managers made sense and meaning of their unique situation during the pandemic.

#### Optimism

A common trait among the managers was their positive mindset during this uncertain time. They described that their businesses were put on hold for a while in the beginning of the pandemic and that there was some uncertainty about what the future would hold. Despite the uncertainty they remained optimistic and believed that the situation would be resolved and that there was still a demand for their services. Several managers, both in Sweden and Norway, stated that they had been through crises in the past that had affected their businesses but that they were accustomed to the ups and downs. The knowledge that they had come through hardships in the past, and managed them well, strengthened their expectation about succeeding this time too.

*“You learn to knock off that feeling of “oh, wow, what are we going to do next month”. You’ve kind of learnt not to care so much about it, it usually works out. You have to have a little attitude. (…) There’s a need for my services and markets.” (IP 4)*

#### Proactivity

Instead of seeing the challenges imposed by the pandemic as a burden, the managers embraced them and focused on finding meaning in them and using this knowledge for the future. For example, the managers from both countries reviewed their offerings and portfolios to decide which services or products to deliver after the pandemic. For some people it was important to have a financial buffer for potential future crises. This increased their feeling of control and reduced the likelihood of experiencing stress. For many managers the pandemic acted as a learning opportunity through which they became aware of the need to be flexible and to have the assets needed to cope with future changes. *“We have spent quite a lot of time trying to improve our basis for future deliverables*, *and reviewed our offering*, *reviewing our portfolio*. *All of that*, *really*. *I mean*, *how*… *what should we deliver after this pandemic*? *[…] I mean*, *when it comes to these extremely drastic events*, *it really becomes a*… *You get somewhat of a breathing space*, *to really think about your position for the future*, *instead of just keeping going*.*” (IP 2)*

In summary, this category revealed that the managers had a positive mindset, and saw the pandemic as an opportunity to stop and think about their future position.

### Manageability

The third category, manageability, identifies the extent to which the managers perceived that they had requisite resources to cope successfully with the challenges, and the extent to which they invested time and energy in finding solutions to these. The category is built on the general resistance resources *problem-solving* and *flexibility and cooperation*, which show how they managed new situations and digital solutions during the pandemic.

#### Problem solving

In some industries daily work proceeded as normal except for the need to follow general restrictions and take extra care in maintaining good hygiene. In other industries a dip was experienced at the beginning of the pandemic and the demand for services dropped from one day to the next. However, these managers were quick to re-adjust and try to find new business ideas and markets in order to reach customers. Even though they were faced with an entirely new situation they managed it by identifying the problems and finding solutions to solve them. As an example, one manager switched focus to selling products to private individuals when the company faced decreased demand from restaurants.

*“We started looking at doing things differently and refining the products.” (IP 7)*

The managers were inclined to try and find solutions to problems as soon as they arose, and to do this they had to take on a number of different tasks such organizing, coordinating and being creative. For many managers this was a challenging period, but they handled it with positivity and saw it as a learning opportunity. One female manager stated:

*“There were slightly different tasks to do at the time. It was quite challenging as a leader really, because all of a sudden, I was doing a lot of different things.” (IP 11)*

### Flexibility and cooperation

In order to stay afloat during the pandemic, many small businesses had to undergo a digital transition and hold a lot of meetings and carry out communication digitally due to restrictions. This was generally seen as a positive experience and a common conclusion was that it had made work more flexible. Being able to have a meeting more spontaneously and deal with the situation shortened decision-making times and increased the manageability of the situation for most managers.

*“Yes, we had more meetings in 2020 than we have ever had. That’s because it’s so simple, well, Teams meetings and stuff. “Let’s take an hour now and check in on each other” and so on, we haven’t done that before. It’s almost as if we may have become more effective in some ways, when we’ve made decisions faster.” (IP 7)*

Digital technology reduced the need for physical presence which made it possible to minimize business trips and commuting. The flexibility this brought about allowed people to have more control of their work and private life.

*“Today we run meetings on Teams or other digital platforms, and I don’t have to drive to another city to attend a meeting for two hours. These types of things are a huge plus, so we get a lot more efficiency out of our days. These are effective meetings in digital forums, which everyone is now comfortable with.” (IP 18)*

In spite of being positive to working remotely, a few Swedish managers stressed the importance of meeting in person from time to time to deal with problems and come up with new ideas. They found it harder to be creative and spontaneous in digital forums.

In summary, the manageability category showed that the managers were quick to find solutions to problems that occurred during the pandemic, and that digital technology made work more flexible and shortened the decision-making time.

## Discussion

The way small business managers handle hardship can have a profound effect on their health and work environment. Given the societal and economic significance of small businesses, this is an important group to study [47, 57]. The objective of this study was to explore whether the salutogenic aspect of SOC was experienced by small business managers in Sweden and Norway during the Covid-19 pandemic. Particular attention was paid to the GRR utilised by these individuals in order to successfully meet challenges during the pandemic.

The main finding of the present study is that the managers in both Sweden and Norway had a salutogenic approach to the pandemic, through which they focused on supportive factors and resources, rather than risks and problems [58]. They used their resources effectively and implemented strategies to address the stressors that arose during this period. They clearly handled the pandemic in a way that worked well in their contexts, and it did not have a negative effect on them or their businesses. These findings are in contrast to other recent research in which Swedish managers of small businesses reported increased workload, a low level of mental well-being, sickness presenteeism, and work-family conflict during the pandemic [9, 22]. In addition, a study from the United Kingdom found that small business managers’ working hours, income and subjective well-being decreased during this time. The authors concluded that the resilience of this group was broken when they faced the reality of dealing with unusual events [59]. Previous research has demonstrated that certain professional demands are specific to small business managers compared to salaried workers, including uncertainty, time demands, risk and responsibility [60, 61], and that these may increase the likelihood of experiencing higher stress [20]. During the pandemic work demands have increased, hence it can be argued that the stress levels for many small business managers have also risen [20, 22]. Therefore, finding strategies to tackle stressors that can emerge both in the workplace and in everyday life and during adversity is crucial for an individual’s well-being [62].

Findings from the interviews in this study demonstrate that it was important for the managers to comprehend and manage the challenges during the pandemic in a resourceful manner, and to see the meaningfulness in the situation. Receiving structured information and social support increased comprehensibility during this period. Social support from family, friends and colleagues was mentioned by the majority of the managers in both countries as an important element to help them cope with the pandemic. This is consistent with the results from a large European survey that found that a high proportion of small business managers in Sweden and Norway indicated that their family, friends and other small business managers were willing to give emotional social support during the pandemic [7]. For several of the Norwegian managers, taking part in professional network meetings where they shared and exchanged experiences with other managers enhanced their understanding of the current situation, which is in line with other research [11, 63]. Previous research has also found that SOC is connected to social support [64, 65], and that social support strengthens an individual’s SOC in a crisis situation [66, 67]. A person with a strong sense of meaningfulness tends to take on the challenges of life, tries to solve and deal with problems and search for meaning in them [24]. This is reflected by the managers in this study as they exhibited a strong sense of meaningfulness as they embraced challenges during the pandemic. They focused on finding meaning in the circumstances and identifying how they could take advantage of the knowledge gained for the future. This aligns with previous research that found that ascribing strong meaningfulness to work helps an individual feel more engaged and increases preparedness [68, 69], as well as functioning as a crucial buffer against the encroachment of a crisis on the workplace experience [70]. A strong SOC facilitates engagement in problem-solving strategies which protects individuals from the negative effects of stress [62]. It was clear that the managers in both countries had a great sense of manageability, and that they were quick to re-adjust and try to find new business ideas and markets in order to reach customers. Even though they were faced with an entirely new situation they managed it by identifying problems and finding solutions to solve them. This result is in line with the findings from a large European survey during the pandemic in which almost 40% of the managers reportedly identified new business opportunities during the pandemic [7]. Another study found that small businesses adjusted their business operations to adapt to the challenging times in the form of increased social media presence, procured supplies and the way they served customers. These changes were important for survival of the business during the pandemic [71], which correlates with previous research that found that small business managers can be creative in finding new solutions for workplace improvements, despite limited in-house resources [11, 72].

## Conclusions and implications

The overall conclusion of this study is that the small business managers handled the pandemic in a way that worked well in their contexts, and that the pandemic did not affect their businesses or themselves negatively. A salutogenic approach through which the managers focused on identifying and using GRRs was an important factor in managing stressors and adversity during the pandemic, and appears to have been a crucial factor for handling the pandemic so well. Hence, the concept of salutogenesis may be used as an intervention to foster better health in small businesses both at a personal and an organisational level [73]. Health promotion activities such as identifying and using resources, creating a friendly work environment, and implementing strategies to prevent stress could improve the work experience of managers and make them better equipped to face hardship [73, 74].

Having a feeling of comprehensibility, meaningfulness and manageability of the situation is also important in order to prevent tension developing into stress. To empower people to deal with stressors, reflection aimed at enhancing these three SOC components is important [75]. Activities that successfully empower managers to deal with everyday life stressors may strengthen their SOC, which may subsequently be applied in new situations to combat stressors [75].

For future research, both qualitative and quantitative studies with larger samples are required to gain a better understanding of the salutogenic effects on small business managers and their workplaces.

### Strengths and limitations

The strength of the study is that it included participants from different types of sectors and with various experiences. An additional strength is that the extensive interviews made it possible to deepen the understanding of the pandemic’s effect on small business managers in their specific contexts. Further, to enhance trustworthiness, the categories, codes and interpretations were compared and discussed between the authors, as there is always more than one probable interpretation of the text. In addition, to ensure dependability and confirmability notes on decisions made during the research process, reflective thoughts after interviews, sampling and research materials adopted were saved in order to be able to review the transparency of the research path [76].

Limitations of this work may be that the managers came from a single geographical context in Sweden and Norway and that managers from industries most commonly affected by the COVID-19 pandemic, e.g., transport, tourism and hospitality weren’t included. The questions in the interview guide had a health promotion perspective, and were not specifically based on SOC, which can be considered a limitation. During the analysis process it was noticed that SOC could add value in the analysis and yield a salutogenic perspective. Hence, the study is of a more descriptive nature. Furthermore, the use of the SOC and its core components of comprehensibility, meaningfulness and manageability as an analysis tool might have implied a potential neglect of other categories of importance to small business managers’ ability to handle the pandemic. However, the pre-defined categories based on the SOC were not used to guide data collection or probe questions during the interviews, they were only applied during the analytical process.
